# Epigenetic Alterations in Triple-Negative Breast Cancer—The Critical Role of Extracellular Matrix

**DOI:** 10.3390/cancers13040713

**Published:** 2021-02-09

**Authors:** Vasiliki Zolota, Vasiliki Tzelepi, Zoi Piperigkou, Helen Kourea, Efthymia Papakonstantinou, Maria-Ioanna Argentou, Nikos K. Karamanos

**Affiliations:** 1 Department of Pathology, School of Medicine, University of Patras, 26504 Rion, Greece; btzelepi@upatras.gr (V.T.); ekourea@upatras.gr (H.K.); 2 Biochemistry, Biochemical Analysis & Matrix Pathobiology Research Group, Laboratory of Biochemistry, Department of Chemistry, University of Patras, 26110 Patras, Greece; zoipip@upatras.gr (Z.P.); n.k.karamanos@upatras.gr (N.K.K.); 3 Foundation for Research and Technology-Hellas (FORTH)/Institute of Chemical Engineering Sciences (ICE-HT), 26110 Patras, Greece; 4 Department of Gynecology and Obstetrics School of Medicine, University of Patras, 26504 Rion, Greece; fayigor_pap@hotmail.com; 5 Department of Surgery, School of Medicine, University of Patras, 26504 Rion, Greece; marianna.argentou@gmail.com

**Keywords:** breast cancer, epigenetics, ECM, EMT, DNA methylation, histone modification, miRNAs, lncRNA

## Abstract

**Simple Summary:**

Current data suggest that epigenetic alterations are involved in the initiation and subclonal evolution of breast cancer. During cancer progression, the extracellular matrix undergoes significant structural alterations and the epithelial–mesenchymal transition is induced. These events among other processes are closely related with the epigenetic modifiers. Triple-negative breast cancer is an aggressive molecular subgroup characterized by genomic complexity and limited therapeutic options. Recent knowledge indicates that matrix alterations in triple-negative cancer cells are epigenetically regulated and that matrix-associated events collectively increase tumor cell survival and resistance to therapy. Thus, approaches for targeting tumor microenvironment and epigenetic pathways, alone or in combination, represent potential therapeutic strategies. The present article aims to highlight the most important epigenetic regulation of extracellular matrix alterations in triple-negative breast cancer in an effort to give perspectives for future design and implementation of diagnostic and therapeutic suggestions.

**Abstract:**

Triple-negative breast cancer (TNBC) is an aggressive subgroup of breast cancer characterized by genomic complexity and therapeutic options limited to only standard chemotherapy. Although it has been suggested that stratifying TNBC patients by pathway-specific molecular alterations may predict benefit from specific therapeutic agents, application in routine clinical practice has not yet been established. There is a growing body of the literature supporting that epigenetic modifications comprised by DNA methylation, chromatin remodeling and non-coding RNAs play a fundamental role in TNBC pathogenesis. Extracellular matrix (ECM) is a highly dynamic 3D network of macromolecules with structural and cellular regulatory roles. Alterations in the expression of ECM components result in uncontrolled matrix remodeling, thus affecting its ability to regulate vital functions of cancer cells, including proliferation, migration, adhesion, invasion and epithelial-to-mesenchymal transition (EMT). Recent molecular data highlight the major role of tumor microenvironment and ECM alterations in TNBC and approaches for targeting tumor microenvironment have recently been recognized as potential therapeutic strategies. Notably, many of the ECM/EMT modifications in cancer are largely driven by epigenetic events, highlighting the pleiotropic effects of the epigenetic network in TNBC. This article presents and critically discusses the current knowledge on the epigenetic alterations correlated with TNBC pathogenesis, with emphasis on those associated with ECM/EMT modifications, their prognostic and predictive value and their use as therapeutic targets.

## 1. Introduction

Triple-negative breast carcinoma (TNBC), is an aggressive molecular subgroup of breast cancer characterized by the absence of estrogen receptor alpha/progesterone receptor (ERα/PR) expression and HER2 (ER/PR/HER2-negative) [1]. TNBCs account for 9–20% of breast carcinomas [1] and are more common in younger age groups, in African–American and Hispanic populations, and in *BRCA* mutation carriers. These tumors are of high histologic grade, and harbor genomic instability and *TP53* mutations [2,3].

Following the identification of the major intrinsic molecular subtypes of breast cancer (i.e., luminal type A, luminal type B, TNBC, HER2+) [4], additional molecular TNBC subtypes were identified, such as the claudin-low subtype, characterized by an epithelial-to-mesenchymal transition (EMT) phenotype [5] and the apocrine subtype, which exhibits activation of the androgen receptor (AR) pathway [6]. Lastly TNBCs were stratified into basal-like 1(BL1), basal-like 2 (BL2), luminal AR (LAR), and mesenchymal (M) [7]. TNBC molecular subgrouping clearly highlights the major involvement of acquisition of mesenchymal properties as well as interactions among extracellular matrix (ECM) and cancer cells, in the invasive capability of TNBC cells.

Structural alterations of the ECM network have been associated with breast carcinogenesis, propagation and progression. Matrix metalloproteinases (MMPs) along with heparanase (HPSE), degrade the ECM components, facilitating breast cancer cell motility, invasion and metastasis [8]. Cross-linking enzymes (i.e., LOX) facilitate collagen maturation, stiffen the matrix, as measured by elastography, and promote breast tumor invasion [9]. ECM components through PI3/AKT, ERK1/2, JNK, Src and JAK2/STAT5 signaling pathways facilitate cancer cell proliferation, expansion, nutrition and escape from growth restriction factors [8].

Altered and hardened ECM also induces EMT and promotes chemoresistance [10]. EMT defines the biological process by which epithelial cells lose their adhesion properties (i.e., E-cadherin and adherens junctions) and gain mesenchymal characteristics (N-cadherin, vimentin) that provide cell detachment and motility [11]. EMT mediated by TGF-β, Notch and Wnt signaling pathways activates the Snail, Slug, ZEB and Twist transcriptional factors [11]. There is growing evidence that altered expression of ECM components induce ΕΜΤ process and novel ECM/EMT interactions have recently been identified. MMPs are the primary driver of adherens junction degradation, whereas remodeling of actin cytoskeleton is driven by ERM proteins (i.e., ezrin, radixin and moesin) which interact with CD44, a cell surface receptor for hyaluronan (HA) and versican [12]. Cellular interactions with the ECM-associated proteins (SPARC, SERPINE) also activate EMT. Recent evidence suggests that tumor-derived extracellular vesicles (EVs) or EVs secreted by cancer associated fibroblasts (CAFs) in the tumor microenvironment play a fundamental role in triggering EMT, tumor invasion, and metastasis [12]. Mechanical signals from ECM (i.e., rigidity, cell matrix adhesion, cell geometry, and cytoskeletal tension) can also provoke EMT through YAP/TAZ signaling [12].

Accumulating data suggest that ECM/EMT pathways in cancer are affected by epigenetic events such as DNA methylation and histone modification. The central role of epigenetics in the regulation of EMT-associated cancer initiation and progression has been reviewed recently [13]. E-cadherin gene (CDH1) is silenced in many types of human cancer, including breast carcinomas, by hypermethylation at CpG islands of the E-cadherin promoter in collaboration with histone deacetylation by HDAC1 and HDAC2 [13]. Many other genes involved in cell polarity (e.g., genes coding for CADM, MYO1A, MPP3, FOXF2) are also silenced by DNA methylation, including breast cancer [14]. Histone methylation is also involved in EMT regulation as it has been shown that the H3K4/K9 demethylase LSD1 can serve as either an activator or a repressor of the ΕΜΤ-associated gene transcription, in a substrate-specific manner [13].

During cancer development, polycomb complex proteins also drive malignant transformation through EMT. It has been shown that certain PRC2 subunits target CDH1, whereas BMI1 through linking to Twist is essential for EMT initiation and the acquisition of the cancer stem cell (CSC) phenotype [13]. B-catenin directly interacts with EZH2 of the polycomb group (PcG), resulting in the enhancement of gene transactivation by the Wnt signaling pathway [14].

Recent research highlights the critical role of microRNAs (miRNAs) in ECM metabolism and the ECM/EMT process. Various ECM effectors are regulated by miRNAs. For instance, HA synthesis mediated by HA synthases (HASs), mainly HAS2, is regulated by miRNAs and chromatin modifications. The long noncoding RNA, HAS2-AS1, induces transcription of HAS2 by promoting O-GlcNacylation and also acts as competing endogenous RNA to sequester miR-7, miR-10b, and let-7 binding [15]. Extracellular vesicles (EVs), in particular exosomes contribute to the process by transferring ECM-degrading enzymes, growth factors, cytokines and miRNAs among the cells [15]. Fibronectin is also indirectly regulated via microRNAs, as exemplified by the case of miR-200b in renal fibrosis and miR-7 in breast cancer [16]. It is worth noticing that upregulation of miR-10b in breast cancer and endometriotic cells results in a direct targeting and downregulation of the cell membrane proteoglycan syndecan-1 [15]. Enzymes that mediate ECM remodeling are also regulated by miRNAs. For example, miR-10b promotes invasion by targeting the transcription factor HOXD10, resulting in upregulation of MMP-14 and urokinase-type plasminogen activator (uPA) receptor (uPAR) in breast cancer cells, thus modulating the proteolytic milieu [16].

ΜiR-200 family is also a critical regulator of EMT and CSCs through ZEB1, ZEB2 and BMI1 expression. Loss of expression of the miR-200 has been found in invasive breast cancer cells and breast CSCs [17]. Furthermore, major PcG proteins such as enhancer of zeste homolog 2 (EZH2) can inhibit miR-200 expression to induce ZEB1 and ZEB2 expression, implying a critical loop between PcG, miR-200 and EMT-TFs [17].

**Figure 1:**
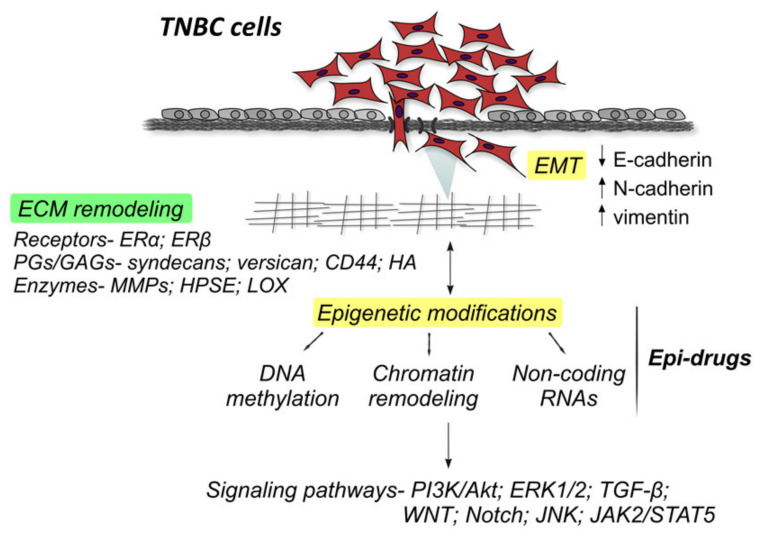
The extracellular matrix (ECM) components that participate in triple-negative breast carcinoma (TNBC) progression include among others major epithelial-to-mesenchymal transition (EMT) biomarkers (E-cadherin, N-cadherin, vimentin, TGF-β etc.), matrix enzymes (matrix metalloproteinases (MMPs), heparanase (HPSE), LOX, HASes, etc.), proteoglycans/glycosaminoglycans (syndecans, CD44, HA etc.), miRNAs (let-7, miR-7, miR-10b, miR-27a, miR-145, miR-200, miR-203 etc.) and estrogen receptors (ERα/β), that affect the activation of signaling pathways critical for the control of TNBC cell properties (PI3K/AKT, ERK1/2, JNK, Src, JAK2/STAT5, WNT, Notch etc.).

TNBC cancer cells characteristically behave as if they have acquired EMT and breast cancer stem cell properties [18] (Figure 1).

They express features of tumor-initiating CSCs such as a CD44^hi^CD24^lo^phenotype which confers resistance to cancer therapeutics and enhances metastatic capacity [19] CD44+ tumor cells are able to bind HA through CD44 and HA–CD44 interaction promotes spheroid tumoral formation with growth and self-renewal capabilities. HA–CD44 binding also induces CD44 translocation to the nucleus and activation of LOX transcription. LOX, in turn, stimulates the transcription of Twist, a major EMT marker [12]. HA-CD44 interaction is also correlated with the transcription of stem cell markers (Nanog/Oct4/Sox2) as well as microRNA (miR-21, miR-10b, and miR-302) and long non-coding RNA (lncRNA UCA1) signaling [20].

Based on these data, it is clearly demonstrated that the network of actions that involves ECM, EMT and stemness is epigenetically regulated and that collectively increases tumor cell survival (Figure 1). Thus, approaches for targeting tumor microenvironment and epigenetic pathways or combinations of them represent potential therapeutic strategies [21].

## 2. Epigenetic Alterations in TNBC: ECM/EMT Interplay

### 2.1. DNA Methylation

**Figure 2:**
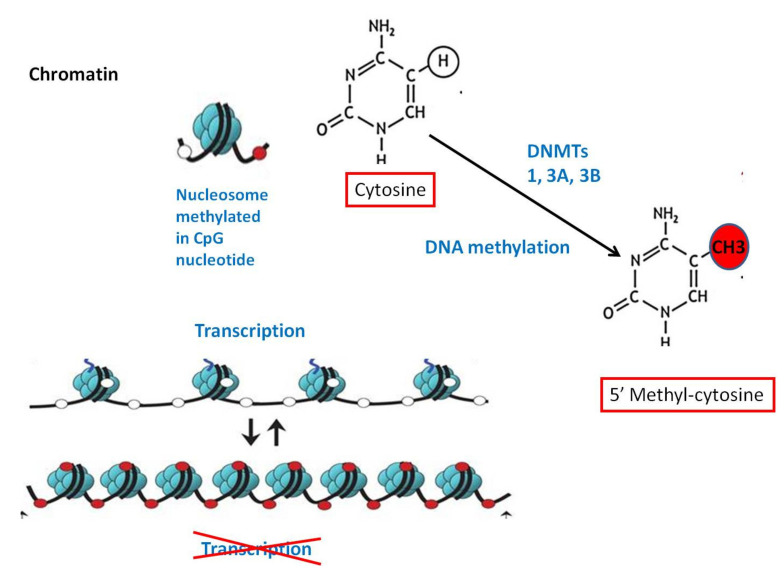
DNA methylation (of the cytosine pyrimidine ring catalyzed by the enzyme methyltransferase) is an “epigenetic switch” that regulates the balance between “open” and “closed” form of chromatin and ultimately resulting in gene silencing.

DNA methylation is one of the best described epigenetic events. CpG islands represent short DNA sequences that are GC-rich, predominantly displaying a non-methylated state. DNA methylation changes in cancer mediated by DNA methyltransferases (DNMT1, 3a and 3b) impact the global structure of heterochromatin and contribute to gene expression alterations. Approximately 70% of commentated gene promoters are associated with CpG islands whose methylation status generally correlates with transcriptional activity [22] (Figure 2).

DNA methylation patterns in TNBCs show many similarities with that of other breast cancer subtypes [23]. For instance, hypermethylation is located in CpG islands and shores, while hypomethylation occurs globally across intragenic regions [23]. However, although the number of methylated CpG islands is similar in TNBC and non-TNBC tumors, the genes that are methylated are different among the different tumor subtypes [24].

The methylation profile of TNBC tumors has been defined by the methylation of five genes (CDKN2B, CD44, MGMT, RB and p73) plus the non-methylation of 11 genes (GSTP1, PMS2, MSH2, MLH1, MSH3, MSH6, DLC1, CACNA1A, CACNA1G, Twist1 and ID4) and it has been concluded that the genomic instability in TNBC is probably acquired by other pathways rather than the methylation of MMR genes [25]. A multi-platform dataset that describes genome-wide assessment of DNA methylation (assessed by Illumina HM450K BeadChip, GSE78751), gene expression (assessed by microarray, GSE61723) and miRNA expression (assessed by microarray, GSE38167) in primary TNBCs, normal adjacent tissues and lymph node metastases, promises to bring to light innovative markers and pathways involved in TNBC progression and metastasis [26].

Intriguingly, a link between DNA methylation and ECM/EMT pathways has been identified. Whole genome DNA methylation analysis in a TNBC cohort including matched lymph node metastases, identified altered novel methylation changes in 18 genes associated with lymph node metastasis and validated the majority (12 out of 18 genes) of them. Notably, most of these genes have a known connection to EMT [27]. Recently, methylation of 313 CpGs, corresponding to 191 genes was observed in TNBC, and it was reported that ECM organization and cell proliferation are the primary characteristics driving breast cancer subtyping [28].

TNBC was recently associated with distinct expression patterns of the two-pore domain potassium channel, and overexpression of both KCNK5 and KCNK9 appeared to be functionally related to hypomethylation of CpG loci [29]. Furthermore, in MDA-MB-231 cells MYC recruited DNMT3A to the miR-200b promoter, resulting in CpG island hypermethylation, followed by miR-200b repression and silencing, thus promoting EMT and mammosphere formation of TNBC cells [30]. A recent review summarized the epigenetic alterations that influenced the expression of EMT-associated genes in TNBC cells [31]. Regarding specific genes, epigenetic silencing of the metastasis suppressor gene CREB3L1 by DNA methylation is prevalent in TNBCs and associated with high grade metastatic breast cancers with poor prognosis [32]. DNA methylation is also involved in the downregulation of breast cancer metastasis suppressor gene 1 (BRMS1) in TNBCs and demethylation was found to inhibit the invasion of breast cancer cells [33]. Methylation of the promoter of dual-specificity phosphatase 1 (*DUSP1*) gene which mediates the dephosphorylation of MAPK, in peripheral blood leukocytes was suggested as a risk factor for TNBC. Further investigations are needed to confirm the reported association of environmental factors, such as fruit and soybean intake, irregular menstruation, and ER/PR status, with *DUSP1* methylation in breast tumor DNA [34].

Although the above data appear promising, there is considerable variation among the results of DNA methylation patterns in TNBC, highlighting the difficulties in drawing meaningful conclusions. Different unbiased studies show major differences concerning the examined CpG sites, the methylation detection methods, and the patient populations and tissues examined and this precludes clinical application of the results.

### 2.2. Histone Modifications

**Figure 3:**
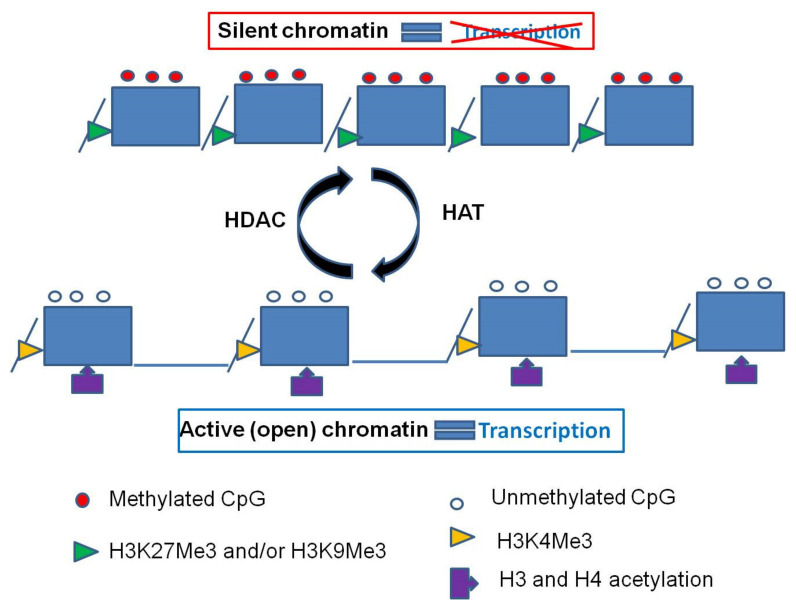
A: CpG islands of actively transcribed genes are unmethylated and have trimethylation at histone H3lysine residue K4 (H3K4Me3) and acetylation on lysine residues at histones H3 and H4. B:Methylation of the CpG island is frequently associated with trimethylation of lysine residue K27 or trimethylation of lysine residue K9 at histone H3, coupled with loss of the active marks H3K4Me3 and acetylated H3 and H4.

Histone modifications are covalent posttranslational alterations (methylation, acetylation, phosphorylation, ubiquitination, sumoylation, etc.) of N-terminal tails of histone proteins that alter the chromatin structure and regulate gene transcription [35] (Figure 3).

Identifying major histone alterations in TNBCs as well as the principle genes influenced by these modifications has been the subject of recent studies with ambiguous results. Varying levels of bulk histone acetylation and methylation have been identified among breast carcinoma subtypes. Moderate to low levels of lysine acetylation (H3K9ac, H3K18ac, and H4K12ac), and lysine (H3K4me2 and H4K20me3) and arginine methylation (H4R3me2) have been observed in carcinomas of poorer prognostic subtypes, including basal-like and HER2-positive tumors [35].

Research has also focused on the potential diagnostic and prognostic role of histone modification factors associated with EMT in TNBC. The differences between breast carcinoma subtypes are further highlighted by the finding of an increase in H3K4ac and H3K4me3 marks in the MDA-MB-231 metastatic cell line compared to the MCF7-mature luminal cell line [36]. In MCF-7 cells, H3K4ac appears to participate in the ER signaling pathway, while H3K4ac together with H3K4me3 were more reflective of the EMT-related genes that promote EMT-mediated signaling cascades in the TNBC cell line, MDA-MB-231 [36].

Recently, the LONESTAR consortium data provided a comprehensive resource for histone alteration profiles and transcription states across the five molecular subtypes of breast cancer, including two triple-negative subtypes, the TNBC-Claudin low, and the TNBC-Basal [37]. Thirteen cell lines, including four TNBCs (MDA-MB-231, MDA-MB-436, MDA-MB-468, and HCC1937) were profiled for eight key histone modifications—H3K4me1, H3K4me3, H3K9me3, H3K9ac, H3K27me3, H3K27ac, H3K36me3, and H3K79me2. The basal-like and claudin low subtypes displayed distinct H3K36me3 patterns. Active promoter and transcription signatures and RNA-Seq identified Actin Filament Associated Protein Antisense RNA 1 (AFAP1-AS1), an antisense lncRNA, as a TNBC specific gene, marked by active histone modifications, such as H3K4me3 and H3K79me2. Exclusive expression of AFAP-AS1 in TNBC cells was further confirmed by RT-qPCR, and was suggested to possibly promote proliferation, migration, or invasion of cancer cells by facilitating EMT, through downregulation of E-cadherin and elevation of mesenchymal markers, such as vimentin, N-cadherin, Slug and Snail. The identification of AFAP1-AS1 as a potential TNBC specific gene and novel molecular target for future development of TNBC therapies has been suggested [37].

Recently, a novel epigenetic pathway that links claudin transcription to breast cancer metastasis was identified. Brahma (BRM) is a chromatin remodeling protein, directly bound to the promoter region of claudin genes via interacting with Sp1 and activates transcription by modulating histone modifications [38]. BRM was found downregulated in MDA-MB-231 breast cancer cells and in high grade human breast cancers and BRM suppression was accompanied by the loss of a panel of claudins in breast cancer cells [38].

Histone methyltransferases (HMTs) catalyze the methylation of lysine and arginine residues. Four HMTs (*ASH1L*, SETDB1, SMYD2 and *SMYD3*) showed mRNA amplification in BL breast cancer while the mRNA levels of eight HMTs (*EZH1*, *SMYD3*, *EHMT1*, *SETD7*, *PRDM4*, *SETD3*, *SETD1B*, and *PRDM6*) were significantly downregulated and twelve HMTs (*EZH2*, *PRDM15*, *PRDM13*, *SMYD2*, *SMYD5*, *SUV39H1*, *SUV39H2*, EHMT2/G9a, *WHSC1*, *SETD8*, *SETDB* and *SETD6*) were significantly up-regulated in BL compared to other subtypes [39]. In the METABRIC study [40], which evaluated downstream signatures for each mutated gene, using the online tool “Genotype-2-Outcome”, *KMT2D*, *SETD1A* and *SETD2* (all included in the lysine methyltransferase pathway), were linked with poor prognosis in invasive breast cancer. Lysine-specific demethylase LSD1 acts by mediating demethylation of H3K4me and H3K9me. LSD1 is highly expressed in ERα-negative tumors with mesenchymal signatures and through Snail activation is responsible for silencing genes encoding E-cadherin, claudins and cytokeratins and correlated with poor survival. These results have prompted investigations into the therapeutic utility of LSD1 inhibitors [14].

Different epigenetic modifications act in parallel to induce an effect. EZH2, a lysine methyltransferase catalyzes H3K27me3 that is associated with heterochromatin formation and silencing of nearby genes associated with cell proliferation, migration and invasion [41]. EZH2 is also capable of recruiting DNMT1 in many cell types to methylate the promoter regions of various genes including tumor suppressors. It has been suggested that EZH2 enforces the silencing of E-cadherin in basal breast cancers and *BRCA1*-deficient tumors, and promotes the mesenchymal cell states of these carcinoma cells by driving H3K27me3 that is associated with the *CDH1* promoter [14]. Evidence also suggests that the transcription factor Sox4 directly regulates the expression of EZH2 and induces the mesenchymal gene program such as vimentin, fibronectin and N-cadherin genes [17]. It has also been shown that G9a/EHMT2, an euchromatin methyltransferase responsible for H3K9me2, is engaged by Snail1, to recruit DNMT1 to CDH1 promoter and induce EMT in TNBC cells [42]. These findings show that a network of epigenetic pathways act synergistically and promote EMT in cancer cells.

Lately it has been shown that cancer stem cells (CSCs) express different DNA and histone methylation patterns compared with non-CSCs. CD44+/CD24−BC stem cells (SCs), from a culture of MDA-MB-231 cells, demonstrated downregulation of both H3K4me2 and H3K27me3, affecting Wnt and GnRH signaling, which resulted in greater invasive and tumorigenic capacities in vivo and in vitro. Breast CSCs are considered important drivers of TNBC aggressiveness although the exact mechanism still remains unknown [43].

Several studies have aimed to reconstitute the normal expression level of epigenetically deregulated genes and reduce TNBC metastatic potential through treatment with specific histone modifiers.

Treatment with HDACis resulted in up-regulation of anti-proliferative, tumor suppressor, and epithelial marker genes in MDA-MB-231 cells and initiated a partial cancellation of the EMT process [44]. Similarly, treatment of TNBC cells that have a mesenchymal phenotype with the HDACi entinostat, resulted in reversal of EMT phenotype, reduction in migratory capacity and reduction in tumor-initiating cells (TICs). More specifically, entinostat was able to reduce the CD44high/CD24low cell population, ALDH-1 activity, as well as protein and mRNA expression of known TIC markers such as Bmi-1, Nanog, and Oct-4. Treatment also significantly reduced tumor formation at the primary site as well as lung metastases [45].

Recently, the antitumor effect of three DNMTis and six HDACis was evaluated using a TNBC cell model and MTT assay, migration and invasion, three-dimensional culture and colony formation assays. Combined treatment both in vitro and in vivo using the most potent DNMTi and HDACi was performed in a panel of breast cancer cell lines. It has been shown that DNMTis and HDACis may reprogram the highly aggressive TNBC cells that have undergone EMT to a less aggressive phenotype. The authors strongly recommended that TNBC is sensitive to epigenetic therapies by reprograming EMT [46].

### 2.3. MicroRNA Expression

**Figure 4:**
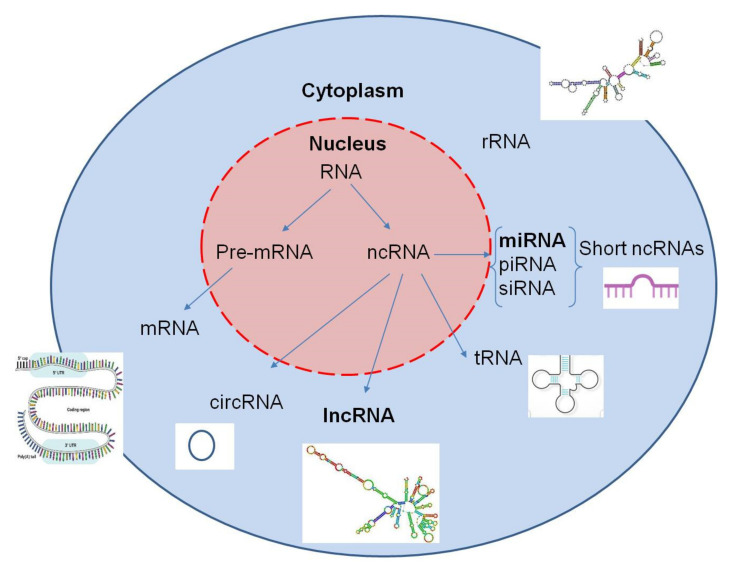
Coding and noncoding RNAs: precursor messenger RNA (pre-mRNA) generates the protein-coding messenger RNA (mRNA). Noncoding RNAs (ncRNAs) include ribosomal RNA (rRNA) and other varieties that are categorized into short and long ncRNAs.

MiRNAs, a type of short ncRNAs, represent RNA sequences that, unlike messenger RNAs (mRNAs), are not involved in gene transcription, but instead they function as a major system of posttranscriptional regulation of DNA expression (Figure 4).

They are considered to be epigenetic regulators in humans with diverse functions in carcinogenesis [47]. Some have been found to be overexpressed in human tumors, known as oncogenic miRNAs (oncomiRs), and others, designated tumor suppressor miRNAs (tsmiRs), are downregulated [48]. Circulating and secreted miRNAs, via membrane vesicles, affect cell–cell communication and cellular metabolic pathways, underscoring the significance of the miRNA–ECM functional relationship in EMT and tumor progression [15] (Table 1).

One of the miRNAs extensively studied in human cancer, miR-10b, is regulated by Twist, MMPs, uPA, and various integrins through direct binging to intronic miR-10b promoter [49]. The direct binding of Twist to the promoter of the miR-10b gene induces cell migration and invasion in metastatic breast cancer [50]. Several miRNAs have been critically linked with processes fundamental to disease progression in TNBC, such as EMT, ECM composition, stemness, invasiveness, migration and metastatic spread, serving as critical TNBC-related biomarkers. In vitro studies have shown that the miR-200 family(miR-200a, miR-200b, and miR-200c) is downregulated in TNBC cells and demonstrated a tumor-suppressive action mediated mainly through downregulation of EMT by targeting ZEB1/2, SIP1 and BMI1 proteins, inhibiting PKC [51] and mediating TGFβsignaling [52]. It has been recently demonstrated that miR-200b upregulation restrains the aggressive phenotype of MDA-MB-231 TNBC cells by inhibiting the invasive potential and migration, followed by ECM remodeling as well as cytoskeletal and major morphological changes [53]. Moreover, lower miR-200b levels have been correlated with poor disease-free survival [54]. Microarray analyses revealed a significant downregulation of miR-205 in TNBC cells induced to undergo EMT [55], similarly to what is described for the miR-200 family.

The miR-200 and miR-205 families directly target the E-cadherin transcriptional repressors ZEB1 and ZEB2 through hypermethylation of their promoters and restrain EMT and cancer metastasis. MiR-203 is also involved in both stemness and EMT in TNBC. It has been shown that miR-203 inhibits tumor cell invasion by post-transcriptionally downregulating the expression of Slug (an EMT-related transcription factor). Moreover, hypermethylation of the miR-203 promoter with ensuing reduced expression of the molecule has been shown in highly aggressive breast cancer cells [15] and upregulation of its expression in TNBC cell lines results in growth and invasion inhibition, enhancement of cell differentiation and reduction in metastatic capacity [56].

MiR-145 is another tumor suppressor miRNA that functions through targeting MMP 11 and Rab GTPase family 27a in TNBC [57]. It also downregulates cell–cell adhesion proteins, resulting in actin cytoskeleton remodeling, breast cancer cell motility reduction and enhancement of apoptosis [15]. MiR-199a-5p also displays a tumor-suppressive role and is downregulated in TNBC. Ectopic expression of miR-199a-5p in MDA-MB-231 cells inhibited the expression of the EMT-related genes CDH1, ZEB1 and Twist, and elevated levels of miR-199a-5p-impaired cell motility, invasiveness and tumor growth in vivo [58]. Research data show that an electrochemical nanobiosensor can be used to measure low concentrations of miR-199a-5p in serum [59].

Forced expression of miR-206 in the mimic-transfected TNBC cells downregulated VEGF, MAPK3, and SOX9 expression levels [60]. The transcriptional loss of miR-211 and the resultant increase in CDC25B expression facilitated increased genomic instability at an early stage of TNBC development [61]. Loss of miR-214 increases the aggressiveness of TNBC via induction of proliferation and EMT, and promotes cell growth by enhancing the PTEN–PI3K/AKT signaling pathway [62]. MiR-223 expression was also down-regulated in CD44+CD24-/low TNBCSCs compared with non-CSCs and miR-223 overexpression resensitized TNBCSCs to tumor necrosis factor-related apoptosis-inducing ligand (TRAIL)-induced apoptosis [63]. Loss of miR-603 expression leads to increase in eEF2K expression and contributes to the growth, invasion, and progression of TNBC [64]. Ectopic expression of miR-378 in MDA-MB-231 cells inhibited Runx1 transcription factor and suppressed migration and invasion, while inhibition of miR-378 in MCF-7 cells increased Runx1 levels and cell migration [65]. Low expression of miR-4417 is significantly associated with worse prognosis in TNBC patients, while overexpression of miR-4417 is sufficient to inhibit migration and mammosphere formation of TNBC cells in vitro [66]. A tumor suppressive role is also exerted bymiR-29b-1-5p [67], miR-211-5p [68], miR-150 [69] and miR-645 [70] in TNBC. Crosstalk between DNMTs, histone modifiers and miRNAs has also been reported [42]. DNMT1 blocks miR-152 expression and subsequently CDH1 mRNA expression, and a DNMT1/miR-152 cyclic feedback loop targeting E-cadherin was proposed in TNBC cells. MiR-340 is a recently recognized tumor suppressor which targets and decreases expression of DNMT1, EZH2 and H3K27me3, leading to promoter hypomethylation and expression of E-cadherin as well as decreased expression of mesenchymal markers (N cadherin, vimentin and fibronectin) in TNBC cells [42]. Mesenchymal TNBC cells (MDA-MB-231 and MCF-7/ADR) are characterized by higher methylation status and elevated DNMT1 and DNMT3A expression than MCF-7 cells. Double-knockdown of DNMT1 and DNMT3A, showed significantly higher miR-200c (a tumor suppressor in breast cancer) expression along with increased E-cadherin and decreased vimentin expression [42].

As far as oncomiRs are concerned, the miR-9 family represents a group of microRNAs upregulated in TNBC [71]. MiR-9downregulates E-cadherin and leads to activation of the β-catenin pathway and upregulation of VEGF. In TNBC, miR-9 was shown to be associated with MYC amplification, tumor grade, metastatic status and poor disease-free survival [51]. MiR-21 is also considered a tumor promoting miRNA, as it has been shown to promote metastasis of breast cancer cells by targeting PTEN, TIMP1, TIMP3, PDCD4, which in turn affect the PI3K/AKT/mTOR pathway [62]. In addition, miR-21 serum levels are linked with TNBC phenotype and familial breast cancer along with lymph node metastasis and higher Ki-67 expression [62]. MiR-221/222, reported to be overexpressed in TNBC, is involved in EMT induction such as E-cadherin downregulation and Slug and Snail upregulation [51].

ERβ, an important mediator of the aggressiveness of ERα-negative breast cancer cells seems to exert its action through regulation of miRNA expression. It has been shown recently that suppression of ERβ in the ERα-negative MDA-MB-231 breast cancer cells decreased the EMT inducer miR-10b and elevated the expression of the tumor suppressor miR-145, thereby inhibiting EMT and provoking major changes in certain matrix components [72] (Figure 1). MiR-10b strongly affects ECM composition, including syndecan-1, MMP-2, MMP-7 and MMP-9 expression and as a result impacts cancer cell migration and invasion. This arrest in the metastatic potential of breast cancer cells suggests the contribution of ERβ in the induction of a more aggressive phenotype in MDA-MB-231 breast cancer cells [72]. A recent review highlights all the microRNAs reported in triple-negative breast cancer cells [73].

### 2.4. Long Non-Coding RNAs

LncRNA HOTAIR, the best studied lncRNA in cancer, has been shown to reprogram chromatin stage and gene expression and promote invasion and metastasis in breast cancer. Elevated HOTAIR expression strongly correlated with lymph node metastasis and with AR expression in a large case series of TNBC patients [74]. It was also postulated that induction of HOTAIR and the consequent repression of PTEN by HOTAIR may neutralize activation of PTEN by homeobox protein HOXA9 in a three-dimensional organotypic culture of the claudin-low breast cancer cells [75].

Several other lncRNAs have recently been associated with ECM/EMT-related molecules in TNBC (Table 1). LINK-A (long intergenic non-coding RNA for kinase activation), is a cytoplasmic lncRNA, which mediates growth factor-dependent HIF1α phosphorylation, stabilization and activation. Both LINK-A expression and LINK-A-dependent signaling pathway activation correlated with TNBC, promoting breast cancer glycolysis reprogramming and tumorigenesis [76]. Small nucleolar RNA host gene 12 (SNHG12), a direct transcriptional target of c-MYC that may promote cell migration by regulating MMP13 expression, has been found to be significantly upregulated in TNBC, its levels correlating with tumor size and presence of lymph node metastasis [77]. A novel metastasis inducing lncRNA which suppresses the KAI1/CD82 metastasis suppressor gene and is upregulated in TNBC has been reported and named Suppressor of KAI1 in Breast Cancer (SKAI1BC) [78].

Two lncRNAs (Airn and PVT1) were shown to regulate TNBC tumorigenesis through opposing actions on the β-catenin signaling pathway [79,80]. Another lncRNA, called LINC01638, maintained the ΕΜΤ traits and the CSC-like state of TNBC cells, through Twist1 expression. LINC01638 knockdown suppressed tumor proliferation and metastasis both in vitro and in vivo [81] and MIR100HG acts as an oncogene through regulation of p27 [82]. LncRNA AWPPH may promote the growth of TNBC by up-regulating the frizzled homolog 7 (FZD7) [83] and/or interacting with miRNA-21 [84]. Plasma levels of lncRNAPOU3F3 were higher in TNBC patients than in healthy controls and inversely correlated with cleaved caspase 9, suggesting that it may promote proliferation and inhibit apoptosis of cancer cells in TNBC [85].

Several studies show that lncRNAs (HCP, PAPAS and LUCAT1) promote triple negative breast cancer by modulating miRNAs (miR-219a-5p, miR-34a and miR-5702, respectively) [86,87,88]. The prognostic and predictive accuracy of an integrated miRNA-lncRNA signature based on the miRNAs FCGR1A, RSAD2, CHRDL1, and the lncRNAs HIF1A-AS2 and AK124454 has been analyzed and this showed that HIF1A-AS2 and AK124454 promoted cell proliferation and invasion in TNBC cells and contributed to paclitaxel resistance [89].

Recent studies have shown that lncRNAs promote TNBC cells invasion through induction of EMT. LncRNA-ZEB2-AS1 promoted the proliferation and metastasis of MDA231 cells in SCID (severe combined immunodeficiency) mice through upregulation of ZEB2 and, thus, ZEB2-AS1 is regarded as an oncogene in TNBC [90]. LINC01638 prevented c-Myc degradation and transcriptionally activated Twist1 expression to induce EMT [81]. LncRNA DLX6-AS1 contributed to elevation of EMT markers, survival and cisplatin resistance of TNBC cells by modulating the miR-199b-5p/PXN axis in vivo [91].

LncRNAs may also act as tumor suppressors in TNBC cells. LncRNA RMST (rhabdomyosarcoma 2-associated transcript) inhibited cell proliferation, invasion and migration thereby enhancing apoptosis and regulating cell cycle [92]. LncRNA NEF overexpression inhibited the migration and invasion of TNBC cells justifying its role as a tumor suppressor [93]. LncRNA PTCSC3 was downregulated, while LncRNAH19 was upregulated and inversely correlated with PTCSC3levels in TNBC tumor tissues. PTCSC3 overexpression led to downregulation of lncRNAH19 in TNBC cells, while H19 overexpression did not affect PTCSC3 expression. Therefore, PTCSC3 inhibits TNBC cell proliferation by downregulating H19 [94]. TNBC patients with low lncMIR503HG expression had a statistically significant worse prognosis compared with those with high MIR503HG expression, and MIR503HG inhibited cell migration and invasion via modulating the miR-103/OLFM4 axis in TNBC [95]. LncRNATCONS_l2_00002973 correlates with less advanced tumor stage and favorable survival, and inhibits cancer cell proliferation while enhancing apoptosis in TNBC [96]. LncRNA XIST through interference with miR-454 inhibits cell proliferation and EMT in TNBC cells in vivo and in vitro [97].

## 3. Conclusions

Recent advances in the rapidly evolving field of cancer epigenetics have shown extensive reprogramming of every component of the epigenetic machinery in TNBC. In addition, an interplay between cancer cells and ECM, as well as acquisition of mesenchymal properties by cancer cells are identified as primary factors underpinning TNBC progression. Epigenetic mechanisms seem to be of major importance in the regulation of EMT/ECM-related pathways. DNA methylation and histone modifying enzymes promote ECM/EMT alterations in TNBC. Several miRNAs have been implicated in the regulation of ECM-dependent processes and have emerged as a novel mechanism in the pathogenesis and progression of TNBC. TNBC treatment may especially benefit from these advances, considering the limited prognostic or predictive tools and/or therapeutic targets currently existing for this subtype of breast cancer. However, despite remarkable advances, further research is required to uncover the mechanisms which regulate the matrix-associated events in cancer progression and the relationship between epigenetics and ECM components of tumor microenvironment.

The major challenge of translating in vitro or in vivo results into clinical practice still remains. In the coming future, multicentric prospective randomized studies testing anti-epigenetic drugs, probably in combination with ECM/EMT-modifying drugs, are expected to be conducted. There is every reason to believe that epigenetic and ECM-targeted approaches will eventually be used in clinical practice and prove to be efficient for developing new diagnostic and therapeutic strategies.

## Figures and Tables

**Table 1:** Major non-coding RNAs (miRNAs and long non-coding (lnc)RNAs) and their ECM/EMT-related targets.

Non-Coding RNAs	Major Affected ECM/EMT Mediators
***miRNAs***	
miR-7	Fibronectin; interleukin-1β
miR-9	CDH1; β-catenin; VEGF; MYC
miR-10b	HOXD10; uPA/uPAR; syndecan-1; Twist; CDH1; vimentin; fibronectin; Snail2/Slug; IGF-IR; HER2; VEGF; MMP-2,-7,-9,-14
miR-21	PTEN; TIMP1; TIMP3; PDCD4; PI3K/AKT
miR-145	CDH1; vimentin; fibronectin; Snail2; HER2; MMP-2,-9,-11; Rab GTPase family 27a
miR-152	CDH1; DNMT1/DNMT3A
miR-199a-5p	CDH1; ZEB1; Twist
miR-200b	CDH1; vimentin; Snail2/Slug; fibronectin; ZEB1/2; BMI1; MMP-2,-7,-9,-14; Erk1/2; ERα/β
miR-203	Snail2/Slug
miR-205	Laminin gamma 1; CDH1
miR-206	VEGF; MAPK3; SOX9
miR-211	CDC25B; MMP-9
miR-214	PTEN-PI3K/AKT; collagen type IV alpha 1
miR-221/222	CDH1; Snail1; Snail2/Slug
miR-223	CD44; TRAIL
miR-378	Runx1
miR-603	eEF2K; IGF-IR
miR-4417	EGFR; IGF-IR; cyclin D1; CDH1; vimentin; p38 MAPK
***Long non-coding RNAs***	
HAS2-AS1	HAS2
HOTAIR	HOXA9; PTEN; AR
LINK-A	HIF1α; EGFR
SNHG12	MMP-13
SKAI1BC	KAI1
Aim, PVT1	KLF5; β-catenin
LINC01638	Twist; metadherin
MIR100HG	CDK18; WEE1; CCNF; CDKN1B; CDC25A
AWPPH	FZD7
POU3F3	Caspase 9
ZEB2-AS1	ZEB2
LINC01638	c-Myc; Twist1
MIR503HG	Olfactomedin 4; MMP-9

Abbreviations: AR, androgen receptor; EGFR, epidermal growth factor receptor; FZD7, frizzled homolog 7; HER2, human epidermal growth factor receptor 2; IGF-IR, insulin-like growth factor receptor; MAPK, mitogen-activated protein kinase; PDCD4, programmed cell death protein 4; PTEN, phosphatase and tensin homolog; SOX9, SRY-Box transcription factor 9; VEGF, vascular endothelial growth factor.
